# Access improvement in healthcare: a 12-step framework for operational practice

**DOI:** 10.3389/frhs.2024.1487914

**Published:** 2025-01-03

**Authors:** Allen M. Chen

**Affiliations:** Department of Radiation Oncology, Chao Family Comprehensive Cancer Center, University of California, Irvine, CA, United States

**Keywords:** access, quality, delays, health policy, leadership

## Abstract

**Background:**

Access improvement is a fundamental component of value-based healthcare as it inherently promotes quality by eliminating chokepoints, redundancies, and inefficiencies which could hinder the provisioning of timely care. The purpose of this review is to present a 12-step framework which offers healthcare organizations a practical, thematic-based foundation for thinking about access improvement.

**Methods:**

This study was designed based on the Preferred Reporting Items for Systematic Review and Meta-Analysis Protocols (PRISMA-P) statement. A literature search of prospective peer-reviewed publications was undertaken to identify studies pertaining to healthcare access. Articles published from January 2014 to January 2024 were included. An interpretive synthesis was then presented.

**Results:**

A total of 469 peer-reviewed studies were identified. The most common diseases analyzed were related to general medicine/family practice (*N* = 75), surgical care (*N* = 51), health screening (*N* = 30), mental health (*N* = 27), cardiovascular disease (*N* = 17), emergency room/critical care (*N* = 15), and cancer (*N* = 7). The remaining 247 studies (53%) did not specifically report on any specialization. The core themes could be broadly categorized into the following: workforce adequacy, patient experience, physical space utilization, template optimization, scheduling efficiency, process standardization, cost transparency, physician engagement, and data analytics. Sixty publications (13%) focused at least in part on equity issues, structural racism, and/or implicit bias; and 25 publications (5%) addressed disparities in education, training, and/or technical literacy. Seventy-three publications (16%) focused either completely or in part on digital health as a means of access improvement.

**Conclusion:**

Based on this systematic review, a 12-step thematically based framework for approaching access improvement in healthcare was developed.

## Introduction

As healthcare organizations grapple with an aging population, escalating costs, and the rapid proliferation of new scientific discoveries, there is arguably no more important issue than access. Indeed, access to healthcare—defined by the National Academy of Medicine as “the timely use of personal health services to achieve the best health outcome—” is central to quality of care, profoundly impacts the patient experience, and influences health outcomes (1). From a practical standpoint, access can best be described as the ability of patients to obtain the care and services they expect when they need them. This critical concept spans the entire healthcare continuum, encompassing everything from making an initial appointment to completing treatment and being appropriately followed thereafter. However, due to the sheer breadth of stakeholders involved in the healthcare marketplace— providers, insurance companies, government regulatory agencies, health systems, industry partners, pharmacies, among others— the coordination required to optimize access, while maintain quality standards, is extraordinarily complex. It is thus hardly surprising that data from the Agency for Healthcare Research and Quality (AHRQ) continues to show that approximately 15% of adults nationwide cannot access healthcare in a reasonably rapid fashion (2). According to a recent survey from McKinsey and Company, the average wait times, as measured by the number of days elapsed, for new patients to obtain primary care and specialist appointments have risen 30% nationwide since 2014 (3). In fact, wait times today for new adult primary care appointments in large metropolitan markets average almost 30 days and climb to more than 100 days in select markets; for some specialists, the wait times are even longer. Remarkably, nearly 30% of American adults report having no primary care provider, and as of 2022, almost 20% hadn't seen any doctor during the past year (4). Thus, the age-old question persists: what can healthcare organizations do to improve access for patients? The purpose of this review is to present a synopsis of the core issues that contribute to access, focusing on actionable, systems-level interventions that could ameliorate barriers in increasingly resource-constrained environments. The 12-step framework that was developed based on a systematic review of the existing literature presents healthcare organizations with practical, thematic-based foundation for approaching access improvement.

## Methods and materials

This study was designed based on the Preferred Reporting Items for Systematic Review and Meta-Analysis Protocols (PRISMA-P) statement. A comprehensive literature search of peer-reviewed publications was undertaken to identify original peer-reviewed works pertaining to access to healthcare services using a variety of search terms including “access improvement,” “timely,” and “delays.” Given the goal of critically evaluating high-level evidence which could enable the preparation of this review, the focus of this work was on specifically identifying original research reporting on the impacts of access on patient care and/or health outcomes. Reference lists from included articles were cross-checked to identify additional articles. Review articles and papers presented as conference proceedings were excluded. Articles published from January 2014 to January 2024 with full text available on PubMed and restricted to the English language and human subjects were included. The full bibliographies of identified articles were reviewed and irrelevant studies including those focused exclusively on the waiting time while physically in the office were selectively removed. Where individual patients were included in multiple published series, the most complete or recent article was cited. Core themes focused on healthcare access were subjectively devised based on the review of the relevant peer-reviewed literature. An interpretive synthesis of the available publications was then presented focused on presenting the evidence evaluating the role of access in healthcare.

## Results

### Search results

**Figure 1:**
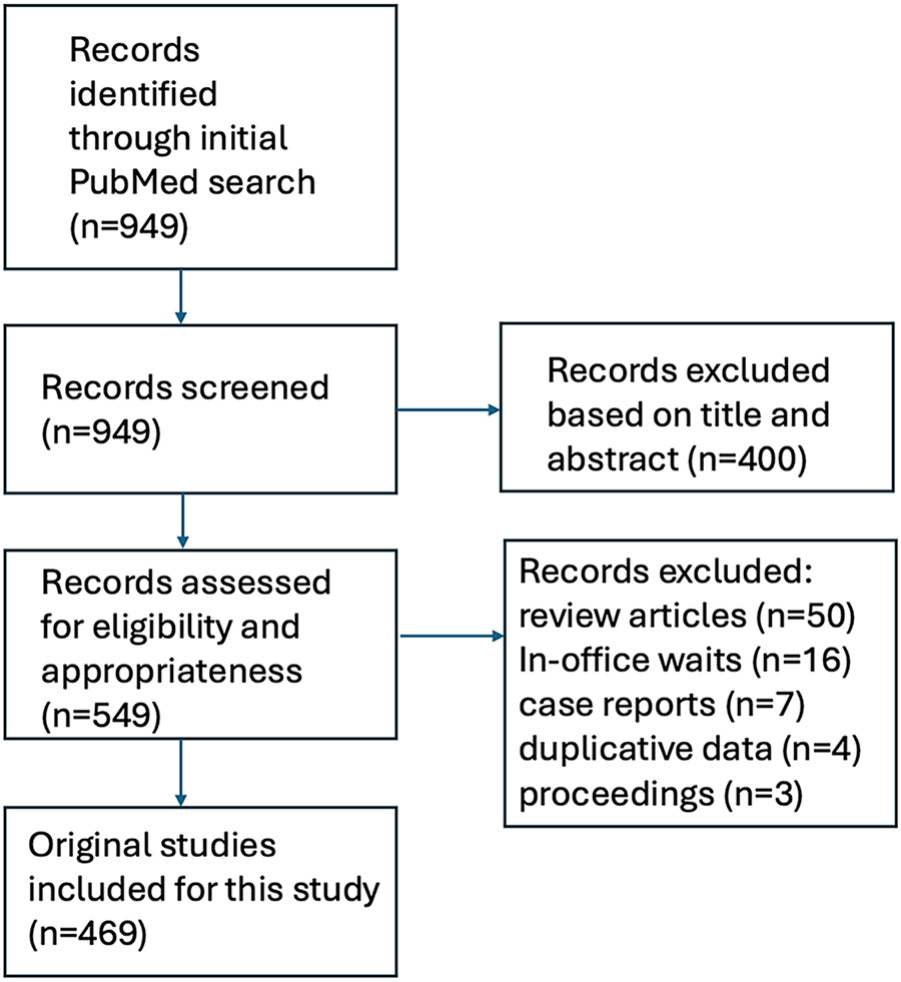
Graphical flowchart of the systematic review.

The initial search yielded 949 articles. After screening of these articles on title and abstract, a total of 549 studies proceeded to full-text screening. Another 80 articles were excluded because they were review articles (*N* = 50), were more focused on in-office delays rather than waits for providers and/or procedures (*N* = 16), was designed as narratives or case reports (*N* = 7), used duplicative data (*N* = 4), or were abstracts only or conference proceedings (*N* = 3). A total of 469 peer-reviewed studies thus were included and formed the basis for this systematic review. Among these 469 works, a total of 398 originated from North America (85%). A schematic illustration of the flowchart outlining the results of the search strategy is shown in Figure 1.

### Identified themes

**Figure 2:**
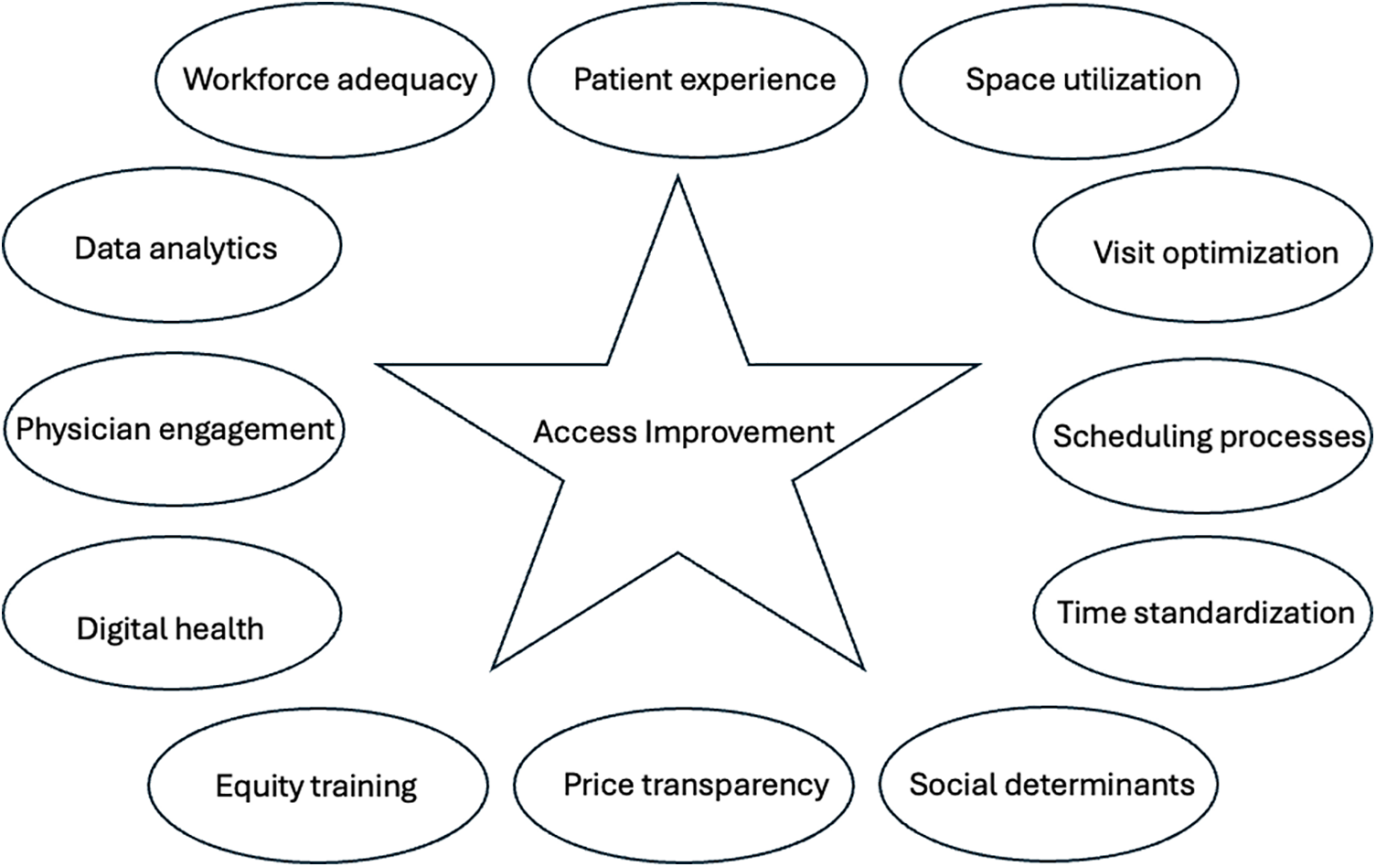
Core themes comprising conceptual framework for access improvement.

The 469 studies that were identified differed significantly in their clinical design, methods, and endpoints. The most common diseases analyzed were related to general medicine/family practice (*N* = 75), surgical care (*N* = 51), health screening (*N* = 30), mental health (*N* = 27), cardiovascular disease (*N* = 17), emergency room/critical care (*N* = 15), and cancer (*N* = 7). The remaining 247 studies (53%) did not specifically report on any specialization. The sample size of human subjects ranged from 33 to 10,550 (mean, 120 patients; median 151; standard deviation ± 95). While some overlap was evident, the core themes could be broadly categorized into the following: workforce adequacy, patient experience, physical space utilization, template optimization, scheduling efficiency, process standardization, cost transparency, physician engagement, and data analytics. Sixty publications (13%) focused at least in part on equity issues, structural racism, and/or implicit bias; and 25 publications (5%) addressed disparities in education, training, and/or technical literacy. Seventy-three publications (16%) focused either completely or in part on digital health as a means of access improvement. The themes comprising this model for access improvement are thus graphically summarized in Figure 2.

## Discussion

Through a systematic review of the evidence, the present study was able to identify core themes that could be used to develop a model for access improvement in healthcare. These could be summarized as follows:

1. **Ensure adequate staffing.** At the most basic level, the ability to satisfy the needs of an expanding patient population depends critically on ensuring that enough providers are in place. While seemingly self-evident, workforce shortages are common bottlenecks in the ability to expand access, as patient backlogs inevitably lead to longer waits. Notably, this supply-and-demand predicament does not just apply to physicians. Studies have shown that a lack of qualified ancillary staff including those related to nursing, front-desk support, and medical assistants can significantly impede workflow— leading to operational delays which can choke patient throughput (5–7). The potential utility of navigators, scribes, and resource specialists has also been demonstrated, as these support staff can absorb much of the mundane, administrative aspects of work, thus enabling physicians to focus on direct medical management (8, 9). Ultimately, a steady workforce allows a practice to run efficiently thereby creating a foundation for improving access. While the geographical disparities in workforce shortage have been well-described, inadequacies in personnel are not limited to rural or inner-city settings (10–12). Indeed, the development of appropriate staffing models is a consideration that all healthcare organizations must pre-emptively address. In the face of shortages, one potential solution is to use locum tenens providers; another is to develop training programs which could serve as a grooming ground and pipeline of talent; yet another is to provide incentives, both financial and non-financial, to prospective providers. All in all, however, there is simply no substitute for solid recruitment. This involves earnestly highlighting the advantages of working at the organization and growing a culture that is inclusive and collegial. Furthermore, it is not merely enough to have just any workforce. As society grows in diversity, the importance of creating a workforce that reflects its varying races and ethnicities is imperative. Indeed, studies across a multitude of industries have shown that environments which actively celebrate diversity and promote inclusiveness are associated with higher levels of employee engagement and consumer satisfaction (13). Finally, the need to pre-emptively address provider burnout is also essential to minimize turnover and to maintain staffing levels (14). Initiatives focused on preserving staff morale and on building workplace wellness can be critical in this regard.
2. **Prioritize the patient experience.** While healthcare is undoubtedly a business—albeit a service-oriented one at that— that fact does not necessarily mean it needs to be impersonal. Nobody, much less patients, enjoy being treated like a number. Central to the development of a personalized approach is the recognition that healthcare is not solely about medical interventions, but also about earning trust and developing meaningful relationships with patients. Efforts to move away from assembly-line, one-size-fits all approach to care are thus urgently needed. Personalization of care truly means focusing on customized healthcare to meet individual patient needs, preferences, and goals. This approach requires healthcare providers to consider the values, backgrounds, and personal circumstances of patients and family when formulating treatment plans. This also means understanding that patients possess lives outside of their health condition and are understandably anxious about the potential inconvenience and disruptions to normalcy that could arise. Unfortunately, a significant proportion of patients acknowledges feeling ignored when discussing their concerns with providers (15). Ultimately, strategies to make the healthcare setting more welcoming and inviting for all will naturally enhance access. However, data continues to show that a considerable proportion of individuals view the healthcare system with skepticism or mistrust, with that number rising dramatically among historically underserved groups (16, 17). Overcoming these perceptions will require thoughtful engagement from a myriad of stakeholders with the goal of ultimately prioritizing timely, equitable, and high-quality care while recognizing that the human connection is at the heart of the patient experience. Although clinical expertise is undoubtedly crucial, a focus on patient-centricity means recognizing that the compassion, kindness, communication, and genuine empathy exhibited by care teams are what leave a lasting impression on patients (17).
3. **Evaluate utilization of physical space.** A critical goal to enhance access is by improving space utilization and proactively identifying and reallocating underutilized clinic rooms. Mismatches between utilization and demand have the potential to create a large amount of waste which can prevent an organization from expanding access. The use of time surveys to prospectively assess overly busy and overly empty junctures during the course of the week. Provider schedules can then be re-arranged to achieve better balance so that rooms are evenly used. By reducing and ramping up usage during peak and off-peak hours, respectively, more efficiency in patient throughout is automatically created without an expansion of resources. The need to identify patient activities that could be potentially moved to other repurposed areas could also make space available for more critical encounters. For instance, the development of observation stations or dedicated changing areas could lead to improvements in the utilization of existing space. In many outpatient settings, the use of a private conference room for patients and family has the ability to free up precious examination rooms for others, especially when consultation visits can take extended periods of time. In situations where space constraints persist, moving lower acuity visits to a virtual format through telemedicine can also be considered. While the need for optimization of existing space is critical to improving access, organizations may ultimately decide that the build out of additional clinic space is the only reliable solution to accommodate the influx of patients and to improve access. In this regard, the utility of urgent care centers and retail clinics have also emerged as access options allowing patients to connect to care outside of a provider's office hours. The establishment of mobile clinics in geographical areas traditionally devoid of healthcare services is another way to address issues with transportation that can impede access.
4. **Optimize existing encounters.** The need to eliminate inefficiencies and redundancies in provider schedules is imperative to enhance access. The utility of an acuity audit of physician schedules was recently demonstrated by our group as we prepared to roll out expanded access services (18). In order to assess the logistical capacity for expanding access using the existing provider pool, an initial review of physician schedules was undertaken to gauge availability. A 3-month prospective audit was conducted of all follow-up patients returning for visits after previously completing treatment with the goal of identifying potentially unnecessary return visits. How many years had it been since the patient completed treatment? How many other providers were the patient currently seeing? At what intervals? These data demonstrated that a high proportion of return patients likely could have been discharged from physician panels and that it was common to observe provider schedules filled with visits where the medical justification was somewhat questionable. For instance, the acuity audit showed that clinic schedules were often squeezed with patients returning greater than 10 years after completing treatment for a condition that was long in remission and/or who were also being seen by a multitude of other specialists at extremely short time intervals. Judiciously identifying patients as such who were seen redundantly and/or unnecessarily was useful in ridding the schedule of backlog. Given the pervasiveness of this phenomenon, it was recommended that audits as such should be repeated periodically. This is because eliminating social visits is imperative to ensure that capacity is optimized to handle new patients with more acute needs. The development of survivorship care templates to transition and off-load patients to primary care physicians can also be of value. Moreover, the acuity audit, by decreasing or eliminating low-value and/or unnecessary visits, strategically allowed the leveraging of existing provider schedules to expand access without the creation of new patient slots. This eliminated any concern among providers that additional work might be created since the goal was to create a leaner schedule. At the same time, it obviated the need to overhaul schedules or to construct any new physician templates; instead, the program enhanced access by optimizing existing space in provider schedules through the identification and elimination of inefficiencies.
5. **Streamline scheduling processes.** Patients frequently cite the act of obtaining and scheduling an appointment as one of the least pleasant experiences in their healthcare journey (19). Pain-points such as automated phone trees, lengthy holds while waiting to speak to a human being, playing phone tag, lack of callbacks, rude customer service— all contribute to frustration for patients and also create massive inefficiencies with respect to access. Throw in the lack of convenient or timely appointments—and it is no wonder that the overall impression of the healthcare system from a user-friendly standpoint can be very low. Thus, the need to modernize scheduling practices is imperative. The centralization of intake coordination to specific teams versed in disease-oriented expertise will make the initial point of contact more friendly and patient-centric. Telephone trees should only be used after hours, if possible, as every attempt should be made to promote voice-to-voice dialogue with patients. Setting expectations to the intake team on how far out a patient could potentially be scheduled will also provide guidance. Once appointments are scheduled, the use of text prompts to patients for confirmatory purposes should be encouraged. Patient pre-registration should also be done prior to the actual appointment date so that the possibility of delays occurring onsite are minimized. The online completion of intake documentation can eliminate bottlenecks leading to patient appointments falling behind schedule, thus making it more seamless to accommodate other patients in the queue. Efforts to eliminate redundancies should be prioritized. For instance, a common complaint from patients is that they are frequently asked to complete intake forms on multiple occasions assessing essentially the same items in paper-pen format typically while in the waiting room—leading to frustrating delays in care. These will have the effect of creating chokepoints in the ability to move patients through the clinic in an efficient manner (20). To expand capacity and offer more convenience to patients, so-called “advanced access” models of scheduling should also be explored (21–23). The possibility of expanding clinic hours to offer appointments that might be more convenient for the working population can also be considered to overcome access barriers. The popularity of same day access—which allows patients to be seen within hours from scheduling an appointment—has also been increasingly demonstrated (24). Lastly, the potential of machine learning tools to predict utilization and to optimize scheduling has increasingly been demonstrated and will likely play a larger role in outpatient management of scheduling the future (25, 26).
6. **Standardize provider templates.** Variability in scheduling practices and in allocated time between providers can stymie any attempt to improve access. Standardizing provider templates promotes transparency and creates a climate where everybody is held to the same level of expectation. For instance, there is no reason why one provider should have an entire hour scheduled for a follow-up visit when another provider has 15 min allocated for the same type of encounter. Similarly, scheduling new patient consultations on the hour (typically for an hour) should be standard. When a provider asks a patient to show up, for instance, at 10:30am, then this results in both the 10am and 11am slots suddenly becoming unusable– throwing a proverbial wrench into the scheduling template and creating inefficiency. Additionally, screening in advance for unauthorized blocks should be conducted. A review of our past templates showed that a significant number of providers had furtively closed off blocks allocated for patient encounters for preparatory time or for charting, despite the fact that the call center had been instructed to schedule patients during these times. These habits created unnecessary stress for the entire intake team and also created bottlenecks which resulted in access delays. To maintain patient flow, double or triple booking of patients should be dissuaded unless absolutely necessary. Last minute cancellations by the provider should also be discouraged, and backup plans should be in place where such situations arise. Lastly, the possibility of aligning compensation in the form of bonuses to citizenship metrics related to access efficiency based on clinic flow and wait times, in addition to patient satisfaction, should be considered.
7. **Address social determinants.** Social determinants of health including factors related to income, education, employment, housing, transportation, and geography, among others, have been shown to contribute significantly to access and moreover, are often pervasive and deeply embedded across generations (27). More recently, as healthcare has become increasingly digitized, varying levels of technical literacy has also created access disparities across the population (28). It is important to recognize that addressing social determinants is not just about checking a box but is about designing with thoughtfulness and intention—and deploying potentially impactful strategies in a pro-active fashion. For instance, models under the Centers for Medicare and Medicaid delivery system are increasingly addressing social needs and implementing community-based preventive programs on a preemptive basis (29). Recently, numerous states required Medicaid managed care plans to screen for and/or provide referrals for social needs, and a recent survey found that nearly all responding plans reported activities to address social determinants of health (30). The availability of dedicated financial counselors should be prioritized, even prior to an actual patient visit. Patients should also be screened to identify at risk groups with respect to housing and transportation. Those living alone have been shown to be at higher risk for poorer outcomes across a variety of different conditions, and social isolation has been well-established to contribute to psychological distress (31). To improve cultural literacy, the use of professional medical interpretation services and multilingual patient education materials can improve cultural responsiveness in healthcare (32). Furthermore, initiatives to promote community-based education focused on prevention and health awareness in historically underserved areas will build engagement and lead to improvements in access. Lastly, it is critical that efforts to improve societal engagement and to address inequities focus both on immediate gaps such as income, housing, transportation, as well as on factors more “upstream” such as early childhood education and wellness including physical activity and violence prevention.
8. **Make price transparency a reality.** With escalating out-of-pocket expenses, healthcare is increasingly cited as unaffordable for a large proportion of the population. Indeed, data from West Health and Gallup poll found that a staggering 29% of adults reported skipping or delaying healthcare for a serious medical condition with that percentage increasing as annual household income decreased (33). It is thus not surprising that price transparency is supported by over 90% of Americans (34). While patient demand for healthcare services generally does not respond in the same manner as consumer demand for other goods in terms of price elasticity, price transparency, in theory, will enable patients to shop for the most effective, lowest-cost healthcare available and drive expenses down as providers compete for market share. The implications with access are immense. After all, when patients are faced with not knowing what they will owe for their care until they receive a bill weeks later, encounters are frequently delayed or skipped altogether. While the Affordable Care Act (ACA) required individual hospitals to make prices transparent by publishing their “chargemasters,” or list prices, for all the services they provide, the resultant effect has arguably increased confusion (35). This is because the unwieldy labyrinth of information published, listing thousands of goods and services posted on thousands of websites is of little practical benefit for patients, who are more interested in out-of-pocket costs. What is needed instead are efforts to provide patients an accurate, personalized breakdown of their estimated financial responsibility prior to being seen. This should be accompanied by initiatives to increase patient engagement, provide real-time assistance with interpreting both outcomes and cost information, compare available treatment and provider alternatives, and couple price information with quality metrics of specific relevance to enable making fully informed decisions. Healthcare organizations have the ability to lead efforts to make price transparency as a means of patient empowerment a reality. This will require engaging a plethora of stakeholders including insurance companies, legislatures, and other government agencies.
9. **Train in bias and equity.** To reduce implicit bias in healthcare, programs to train staff in cultural competency and to create policies that are inclusive and sensitive to the needs of all have the potential to address longstanding disparities faced by disadvantaged groups (36). Providers need to understand that Implicit bias is a pervasive issue in healthcare and that structural racism, defined as a form of social formation embedded within a network of social, economic, and political entities in which groups of people are categorized and hierarchically ordered through a historical process of racialization, can have major impacts on care delivery and patient outcomes (37–39). Notably, nearly half of healthcare workers in the United States have witnessed racial discrimination against patients and say this is a crisis or major problem, according to 2024 survey by the Commonwealth Fund (40). According to findings from another large, nationally represented survey published by the Kaiser Family Foundation in 2023, the percentage of minorities who personally experienced discrimination in healthcare was frequent. In total, approximately 60% of African American adults, half of Native American and Latino adults, and 40% of Asian adults admitted to preparing for possible insults from providers or staff and/or felt they must be careful about their appearance to be treated fairly during health care visits (41). Furthermore, the survey found that patients who experienced discrimination were more likely to have reported feelings of anxiety, loneliness, and depression. Another poll found that greater than half of Black person/persons/people and Hispanic person/persons/people believed that the “healthcare provider is biased against me based on their attitude, words, or actions” (42). Fifty percent of respondents also reported “a healthcare provider assuming something about me without asking me.” As society becomes more multi-cultural, the proportion of patients who are non-English speaking is also expected to increase. This is of practical relevance since the logistical complexities associated with navigating the healthcare system poses particular difficulties for those from disadvantaged and/or vulnerable backgrounds (43). Practical initiatives to address the fears of those who have been historically neglected are critical to even the playing field for all with respect to access improvement.
10. **Invest in digital health.** Digital communication tools, such as electronic patient portals, mobile health apps, telemedicine, AI-based virtual navigators, and online health information resources, have gained significant popularity and are increasingly being integrated into healthcare delivery systems. These platforms offer unique opportunities to enhance patient engagement and to reach a wide range of demographics, regardless of geographic location, socioeconomic status, or educational background— and to bridge access gaps in care. One of the advantages of such technology is that access can be instantaneous and conveniently achieved in the comfort of a patient's own home. Furthermore, the rapid deployment of health-related wearables and mobile technology has contributed to more efficient ways of dynamically monitoring patients for a variety of conditions outside of the hospital. Moreover, increasing attention is being focused on the use of technology to address barriers to access and growth in healthcare. Investigators from Australia recently reported on an innovative trial using the internet of things (IoT), connective devices to assist in the optimization of physical space in the outpatient clinic (44). Using sensor technology to assess real-time traffic and clinic census, the researchers showed the potential of live feedback from the IoT to improve clinical space utilization and to develop organizational strategies for operational improvement. The utility of having a trained online navigator who is available to answer questions and to help triage patients may be beneficial (45). Lastly, digital tools which allow for self-scheduling of appointments in a platform that displays all possible openings will further patient engagement. Given the increasing implemental of digital health platforms to ostensibly enhance the patient-provider relationship, the potential of technology to improve access is just starting to become realized. The advent of the digital age in healthcare has spawned a growing belief that technology will streamline processes and eliminate many barriers to access. However, it must be recognized that digital tools have potential limitations as well. For instance, the use of wearables can contribute to more anxiety leading to a “worried well” phenomenon and also lead to over-diagnosis in certain situations (46). Lastly, the potential of digital technology can also introduce new disparities (“the digital divide”) based on access and utilization to these tools (47–49).
11. **Promote physician engagement.** For many healthcare organizations, prioritizing and/or expanding access will represent a dramatic shift in culture affecting numerous aspects of workflow. Given the potentially disruptive nature of this paradigm, engagement at all levels of the workforce is imperative to its success. This starts with the provider cohort. Without the genuine buy-in of the physicians, an environment of shared purpose and commitment will be difficult to establish. This is especially important since access improvement will often be met with initial skepticism and/or viewed as a fancy marketing gimmick more in line with padding the financial pockets of the organization than with an earnest effort to enhance the patient experience. In this sense, the dangers of underestimating the challenges associated with change, particularly in an environment such as healthcare where habits and processes regardless of their effectiveness are difficult to modify over time. Physicians, after all, are well-known to be individuals of habit and resistant to even seemingly small adjustments in their scheduling customs (50). They can also be fiercely protective of their time. The role of physician champions, key influential leaders who can drive the vision of access improvement, will thus be key to engage all stakeholders on a consistent and visible basis, reminding everybody of the merits of timely care. To further build buy-in, town halls with question-and-answer sessions should be considered to openly discuss issues related to patient access with the goal of encouraging input from all members of the organization.
12. **Harness the power of analytics.** Scorecards and dashboards utilizing a variety of objective metrics are increasingly used to evaluate every aspect of the healthcare environment. Patient access should be no different. Indeed, a slew of key performance indicators are commonly used in practice including access-related benchmarks such as third-next-available appointment, time from referral to appointment, office wait time, patient call handle time, no show percentage, appointment cancellation rate, survey results from patient experience surveys. As previously discussed here within, analytics related to scheduling capacity, space utilization, and health equity have the potential to truly take access metrics to the next level. Given the importance of continuous analysis of data to gauge operational success, it is highly recommended that analytics be acquired prospectively and reviewed on an ongoing basis—so that modifications can be made to procedural elements in real-time. The integration of AI-based tools also has the potential to transform data analytics with respect to access improvement. For instance, the data acquired during routine care can be inputted into machine learning models to create algorithms to assist with patient flow (51, 52). Moreover, studies have shown that AI can predict patients who are at risk for no shows and can assist with scheduling processes by smartly considering such factors as space, provider time, and demand based on historic patterns (53, 54).

## Conclusion

Improving access is a fundamental component of value-based healthcare as it inherently promotes quality by eliminating chokepoints, redundancies, and inefficiencies which could hinder the provisioning of timely care. Yet as healthcare organizations struggle with cost-containment, the question of how to best enhance access remains largely unsolved. Indeed, given the economic, regulatory, and social forces at play in the healthcare marketplace, the critical importance of access in optimizing efficiency is frequently underestimated. Thus, the 12-step framework presented herein offers healthcare organizations a practical foundation for thinking about access improvement.

Healthcare organizations are increasingly recognizing that an investment in access improvement will be rewarded in a multitude of ways. By improving quality of care and prioritizing patient satisfaction, brand loyalty will invariably grow to new levels. It is thus likely that improved access will draw in patients who otherwise may not have entered the system. As such, the potential impact of access improvement on a healthcare organization's financial bottom line should also be recognized. From a system standpoint, improved access can help reduce costs as patients who face barriers accessing care may wait until their condition becomes more severe before seeking treatment. Additionally, access delays can result in patients using the emergency room inappropriately as their point of entry to care contributing to waste across the enterprise. The phenomenon of patients leaving a system entirely and opting to seek care elsewhere due to excessively long waits is also well-known (55). Notably, the missed appointments that consequently arise can lead to excessive vacancies in provider schedules resulting in underutilization of resources and increased expenses. The implications for sustainable business operations in an increasingly value-based healthcare landscape can thus be immense.

Ultimately, access improvement is a powerful driver for patient-first, consumer-centric healthcare. While forecasting the future of healthcare is an imperfect science, especially as the boundaries between technology, medicine, business, public health, and policy become increasingly blurred, maintaining focus on the human element of the patient-provider experience is imperative. How current trends in healthcare will potentially integrate access paradigms into delivery models will be closely monitored in the future.

## Data Availability

The original contributions presented in the study are included in the article/Supplementary Material, further inquiries can be directed to the corresponding author.
